# Comparison of anaesthesia strategies on postoperative nausea and vomiting in laparoscopic sleeve gastrectomy: a randomised controlled trial

**DOI:** 10.1186/s12871-024-02577-8

**Published:** 2024-06-13

**Authors:** Ying Yang, Bucheng Liao, Ruoxi Deng, Liwei Ren, Yongjie Sun, Shaowei Xiong, Xinhai Wu

**Affiliations:** 1 Department of Anesthesiology, Peking University Shenzhen Hospital, No. 1200 Lianhua Street, Futian District, Shenzhen, Guangdong 518036 China; 2 Hubei University of Medicine, Shiyan, Shenzhen, China; 3 Department of Gastrointestinal Surgery, Peking University Shenzhen Hospital, Shenzhen, China

**Keywords:** Laparoscopic sleeve gastrectomy, Bariatric surgery, Postoperative nausea and vomiting, Goal-directed fluid therapy, Total intravenous anaesthesia

## Abstract

**Background:**

Intra-operative anaesthesia management should be optimised to reduce the occurrence of postoperative nausea and vomiting in high-risk patients; however, a single intervention may not effectively reduce postoperative nausea and vomiting in such patients. This study assessed the effect of an optimised anaesthetic protocol versus a conventional one on postoperative nausea and vomiting in patients who underwent laparoscopic sleeve gastrectomy.

**Methods:**

A single-centre randomised trial was conducted at Peking University Shenzhen Hospital from June 2021 to December 2022. Among 168 patients who underwent laparoscopic sleeve gastrectomy, 116 qualified, and 103 completed the study with available data. Patients were categorized into the conventional group (received sevoflurane and standard fluids) and the optimised group (underwent propofol-based anaesthesia and was administered goal-directed fluids). The primary endpoints were postoperative nausea and vomiting incidence and severity within 24 h.

**Results:**

Postoperative nausea and vomiting assessment at 0–3 h post-surgery revealed no significant differences between groups. However, at 3–24 h, the optimised anaesthetic protocol group showed lower postoperative nausea and vomiting incidence and severity than those of the conventional group (*P* = 0.005). In the conventional group, 20 (37.04%) patients experienced moderate-to-severe postoperative nausea and vomiting, compared to six (12.25%) patients in the optimised group (odds ratio = 0.237; 95% CI = 0.086, 0.656; *P* = 0.006). No significant differences were noted in antiemetic treatment, moderate-to-severe pain incidence, anaesthesia recovery, post-anaesthetic care unit stay, or postoperative duration between the groups. While the total intra-operative infusion volumes were comparable, the optimised group had a significantly higher colloidal infusion volume (500 mL vs. 0 mL, *P* = 0.014) than that of the conventional group.

**Conclusions:**

The incidence and severity of postoperative nausea and vomiting 3–24 h postoperatively in patients who underwent laparoscopic sleeve gastrectomy were significantly lower with propofol-based total intravenous anaesthesia and goal-directed fluid therapy than with sevoflurane anaesthesia and traditional fluid management. Total intravenous anaesthesia is an effective multimodal antiemetic strategy for bariatric surgery.

**Trial registration:**

This trial was registered with the Chinese Clinical Trial Registry (ChiCTR-TRC- 2,100,046,534, registration date: 21 May 2021).

## Background

Postoperative nausea and vomiting (PONV) occur in approximately 80% of the bariatric surgical population compared to 40% in the general surgical population [1, 2]. Laparoscopic sleeve gastrectomy (LGS) has the highest incidence of PONV among all standard bariatric surgical procedures [3]. PONV is associated with significantly decreased patient satisfaction, prolonged hospital stays, and increased healthcare costs [4].

Despite intra-operative pharmacological prophylaxis in LSG, more than two-thirds of the patients experience PONV [5]. Multiple factors lead to the development of PONV in these patients, classifiable as patient-, surgery-, or anaesthesia-related factors. The first two categories are challenging to manage in clinical settings. To minimise these risks, anaesthesiologists optimise intra-operative anaesthesia management to reduce the occurrence of PONV in high-risk patients, including opioid-free/sparing anaesthesia technique, avoiding volatile anaesthetics and goal-directed fluid therapy (GDFT) [5–8]. Opioid-free/sparing anaesthesia technique has been shown to reduce postoperative morphine requirements and the incidence of PONV [9, 10]. However, the potential effect of a single intervention (total intravenous anaesthesia or fluid management strategies) in reducing PONV is ambiguous in high-risk patients. We hypothesised that combining these two strategies to optimise the anaesthesia protocol may significantly reduce PONV in patients undergoing LSG.

This randomised controlled trial (RCT) primarily aimed at analysing whether a perioperative optimised anaesthetic protocol (propofol intravenous anaesthesia combined with GDFT and optimised anaesthetic protocol [OAP]) results in a lower incidence of PONV compared to the conventional anaesthetic protocol (CAP) (sevoflurane inhalation anaesthesia combined with conventional fluid therapy and CAP) in patients undergoing LSG.

## Methods

### Study design and ethics statements

This single-centre clinical RCT was conducted at the Peking University Shenzhen Hospital, an affiliated hospital of Peking University located in Shenzhen, Guangdong Province, from June 2021 to December 2022. This study was approved by Professor Tao Wang, the chairman of the Clinical Research Ethics Committee of Peking University Shenzhen Hospital (IRB 2021[036]), and written informed consent was obtained from all participants. Before patient enrolment, this trial was registered with the Chinese Clinical Trial Registry (ChiCTR-TRC- 2,100,046,534, registration date: 21 May 2021).

### Inclusion criteria

Potential participants were screened the day before surgery (or on the preceding Friday for those who underwent surgery on a Monday). Patients with obesity aged 18–65 years who underwent elective LSG were eligible for the study.

### Exclusion criteria

Patients were excluded if they met any of the following criteria: (1) history of severe arrhythmia, myocardial infarction, or cardiac insufficiency (NYHA III/IV); (2) respiratory diseases; (3) hepatic/renal insufficiency; or (4) preoperative use of antiemetics.

### Data collection

Detailed patient information was obtained after recruitment, including baseline demographic data, preoperative medical history, diagnosis at the time of admission, illness severity, and perioperative variables. After obtaining written informed consent, baseline data (demographic data, surgical type, and comorbidities) were collected.

### Randomisation and blinding

A biostatistician not involved in the data management and statistical analyses generated random numbers (at a 1:1 ratio) using SAS software version 9.2 (SAS Institute, Cary, NC, USA) with a block size of 4. The results of this randomisation were sealed in sequentially numbered envelopes and maintained until the end of the study by a study coordinator (Y.Y.) who was not involved in the data collection, perioperative care, or postoperative follow-up. During the study period, consecutively recruited patients received intra-operative OAP or CAP according to random number allocation by the study coordinator (Y.Y.). The investigators (B.L. and R.D.) collected intra-operative data for each recruited patient. The anaesthesiologists and investigators did not communicate with each other regarding the collected patient data. The patients and postoperative investigators were blinded to the group assignment.

### Anaesthetic management

No premedication was administered; solid food and clear fluid intake were allowed until 8 h and 2 h preoperatively, respectively. Non-invasive blood pressure, peripheral oxygen saturation, electrocardiogram, bispectral index (BIS), and body temperature were continuously monitored. After preoxygenation for denitrogenation using the mask, propofol (1–2 mg∙kg^− 1^ total body weight), sufentanil (0.5 µg∙kg^− 1^ lean body weight [LBW]), and rocuronium (0.9 mg∙kg^− 1^ LBW) were administered for anaesthesia induction. Dexamethasone (10 mg i.v.) was administered as a prophylactic antiemetic after endotracheal intubation. The respiratory parameters were set as follows: tidal volume of 8 mL∙kg^− 1^ according to ideal body weight, respiratory rate of 10–12 times∙min^− 1^, and inspiratory-to-expiratory time ratio of 1:2. The respiratory parameters were adjusted to ensure a P_ET_CO_2_ between 35 and 45 mmHg. Anaesthesia was maintained with sevoflurane (1.5–2.5%) and remifentanil (0.08–0.15 µg∙kg^− 1^∙min^− 1^) in the CAP group and propofol (6–8 mg∙kg^− 1^∙h^− 1^ LBW) and remifentanil (0.08–0.15 µg∙kg^− 1^∙min^− 1^) in the OAP group, keeping the BIS between 40 and 60. Sufentanil and rocuronium were administered to maintain adequate analgesia and muscle relaxation levels. Subsequently, 10 mg of azasetron was administered intravenously 30 min before the end of the procedure. For intra-operative fluid management in the CAP group, preoperative fluid deficit was estimated using the 4-2-1 rule based on the patients’ LBW and an 8-h fasting time. After the participants entered the operating room, half of the deficit was administered before induction using lactated Ringer’s solution. The other half was supplemented at the discretion of the attending anaesthesiologist to maintain a mean arterial blood pressure (MAP) > 65 mmHg and a urine volume > 0.5 mL kg^− 1^ h^− 1^. Whenever the MAP decreased to < 65 mmHg, rapid fluid infusion and vasoactive drugs (phenylephrine, ephedrine, or norepinephrine) were administered to maintain MAP at > 65 mmHg. In the OAP group, a pleth variability index (PVI) sensor (RainbowR2-25 a, Masimo Corporation, Irvine, CA, USA) was placed on the patient’s index finger to monitor the PVI continuously. The preoperative fluid deficit was corrected using the same protocol used for the CAP group. Half of the deficit was administered before induction using lactated Ringer’s solution, followed by a continuous infusion of crystalloids (2 mL kg^− 1^ h^− 1^). When the MAP was < 65 mmHg, the vasoactive drugs were promptly administered to maintain a MAP of ≥ 65 mmHg regardless of PVI. When the MAP was ≥ 65 mmHg, fluid responsiveness was considered with a PVI > 13%. Whenever the PVI was > 13% for 5 min, we administered a 250 mL bolus of colloid (hydroxyethyl starch 6%, Voluven®, Fresenius Kabi, Beijing, China). The dose was repeated every 5 min whenever the PVI remained higher than 13%. Subsequently, vasoactive drugs (phenylephrine, ephedrine, or norepinephrine) were administered to maintain MAP > 65 mmHg [11].

### Postoperative prevention of PONV and pain management

When the patient returned to the ward, 10 mg of metoclopramide was administered routinely to prevent PONV. This was not considered an antiemetic treatment. If obvious PONV occurred in the ward, additional 10 mg of metoclopramide was administered as antiemetic remedy treatment. Postoperative pain was treated with intravenous flurbiprofen axetil (50 mg) or parecoxib sodium (40 mg) every 12 h for the first 24 h. Postoperative pain was measured using an 11-point numerical rating scale (NRS). Patients who experienced postoperative breakthrough pain received intravenous tramadol 100 mg.

### Outcome assessments

The primary endpoints were the incidence and severity of PONV within 24 h postoperatively. The secondary endpoints included antiemetic treatment, time-to-first-remedy, incidence of moderate-to-severe pain, and postoperative length of stay (PLOS).

The severity of PONV was evaluated using a 4-point NRS, where a score of 0 indicated no PONV, 1 indicated mild PONV (nausea), 2 indicated moderate PONV (retching), and 3 indicated severe PONV (vomiting) [12].

### Sample size

According to a retrospective analysis, the incidence of PONV in patients in the Peking University Shenzhen Hospital was 75% 24 h after LSG. The multimode intervention was hypothesised to reduce the absolute risk by 50%. With a test level of α = 0.05 and a test efficacy of 1-β = 0.80, each group required 55 patients. Consequently, 5% of the samples were lost, and 58 patients were expected in each group.

### Statistical analysis

Initial data were collected using an Excel spreadsheet and transferred to an SPSS dataset for analysis. The Shapiro–Wilk test was used to analyse the Gaussian distribution of the quantitative data; *P* values < 0.05 were considered non-Gaussian distributions. Gaussian data were defined as mean and standard deviation (SD); non-Gaussian data were presented as median and interquartile range (IQR). The Gaussian data were analysed using the Student’s t-test, and the non-Gaussian data were analysed using the Mann–Whitney U test for significance. Categorical data were analysed using Pearson’s chi-square or Fisher’s exact test. *P* values < 0.05 represented statistical significance.

## Results

### Preoperative parameters

**Fig. 1:**
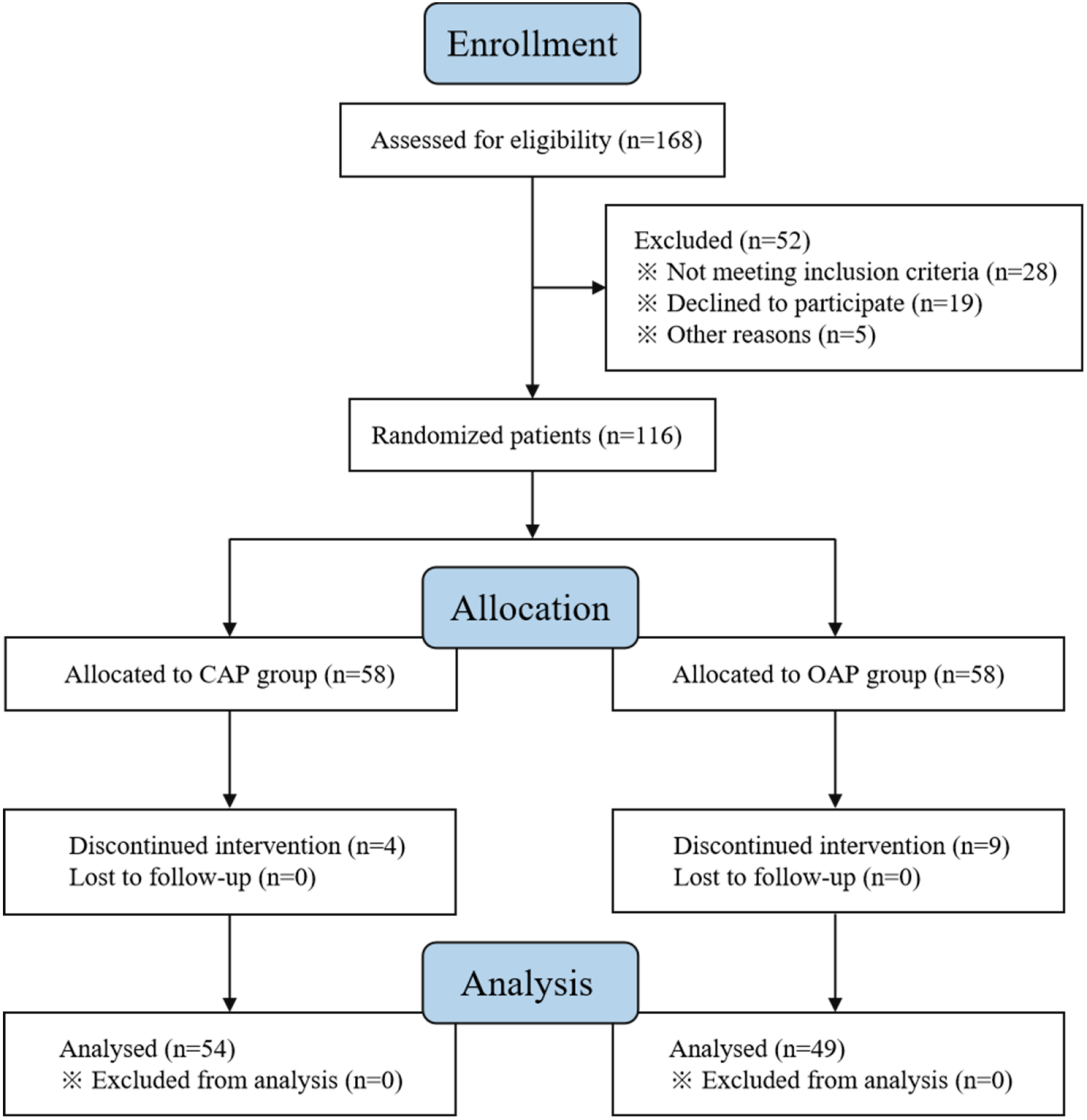
Flow diagram of the participant selection process for the trial. CAP: conventional anaesthetic protocol, OAP: optimised anaesthetic protocol

A total of 116 patients who underwent LSG were included and randomised to receive either CAP or OAP. After randomisation, four patients in the CAP group and nine in the OAP group were excluded from the analysis due to discontinued intervention. No patient was lost to follow-up. Fifty-four participants in the CAP group and 49 in the OAP group completed the study and were evaluated (Fig. 1). The baseline characteristics and comorbidity data of the included patients are listed in Table 1; there were no statistically significant differences between the two groups.

**Table 1:** Baseline characteristics of the participants

	CAP group (*n* = 54)	OAP group (*n* = 49)
Baseline data		
Age (years), mean (SD)	31.65 (6.84)	31.14 (7.61)
BMI (kg m^− 2)^, mean (SD)	36.86 (4.82)	37.52 (5.32)
Female sex, *n* (%)	39 (72.22)	38 (77.56)
ASA score, *n* (%)		
II	52 (96.33)	45 (91.84)
III	2 (3.67)	4 (8.16)
Apfel Score, *n* (%)		
0	1 (1.85)	1 (2.04)
1	7 (12.96)	5 (10.20)
2	17 (31.48)	19 (38.78)
3	20 (37.04)	14 (28.57)
4	9 (16.67)	10 (20.41)
Comorbidities		
Hypertension, *n* (%)	44 (81.48)	38 (77.55)
T2D, *n* (%)	39 (72.22)	37 (75.51)
Hyperlipidaemia, *n* (%)	35 (64.81)	34 (69.39)
OSAS, *n* (%)	15 (27.78)	10 (20.41)
Smoking, *n* (%)	8 (14.81)	7 (14.29)
Motion sickness, *n* (%)	12 (22.22)	11 (22.45)

Continuous data are expressed as means ± standard deviation or medians (interquartile range). Categorical data are expressed as the number of patients (percentage). Statistical significance was tested using the t-test, Mann–Whitney U test, Pearson’s chi-square test, and Fisher’s exact test

ASA, American Society of Anaesthesiologists; BMI, body mass index; CAP, conventional anaesthetic protocol; OAP, optimised anaesthetic protocol; OSAS, obstructive sleep apnoea syndrome; SD, standard deviation; T2D, type 2 diabetes

### Intra-operative parameters

The intra-operative parameters are shown in Table 2. No inter-group differences were observed in the duration of surgery, anaesthesia, awakening, post-anaesthetic care unit (PACU) time, or opioid consumption. Total and crystal infusion volumes were similar between the two groups. However, the volume of colloids in the OAP group was significantly higher than that in the CAP group (0 mL vs. 500 mL, *P* = 0.014). In addition, no differences were observed in blood loss or urine volume. There was no significant difference in the use of antiemetics during surgery or in the PACU.

**Table 2:** Intra-operative variables

	CAP group (*n* = 54)	OAP group (*n* = 49)	*P*
Duration			
Surgery duration (min)	135 (110, 166)	130 (115, 170)	0.861
Anaesthesia time (min)	180 (148, 121)	175 (160, 208)	0.599
Time to extubation (min)	20 (12, 30)	24 (20, 35)	0.183
PACU duration (min)	65 (46, 90)	65 (50, 75)	0.654
Opioid administration			
Sufentanil (ug)	50	50	0.780
Remifentanil (ug)	943.25 (777.38, 1341.52)	958.50 (817.50, 1159.05)	0.947
Tramadol (mg)	0 (0, 0)	0 (0, 0)	0.626
Fluid management			
Total volume (mL)	1600 (1600, 1950)	1600 (1600, 2100)	0.256
Crystalloid (mL)	1600 (1100, 1600)	1500 (1100, 1600)	0.276
Colloid (mL)	0 (0, 500)	500 (0, 500)	0.014
Bleeding (mL)	10 (10, 20)	10 (10, 20)	0.344
Urine (mL)	200 (100, 300)	200 (150, 375)	0.115
Antiemetic			
During surgery, *n* (%)	51 (94.44)	49 (100.00)	0.140
During PACU, *n* (%)	3 (5.56)	2 (4.08)	0.546

Continuous data are expressed as means ± standard deviation or medians (interquartile range). Categorical data are expressed as the number of patients (percentage). Statistical significance was tested using the t-test, Mann–Whitney U test, Pearson’s chi-square test, and Fisher’s exact test

CAP, conventional anaesthetic protocol; OAP, optimised anaesthetic protocol; PACU, post-anaesthetic care unit

### Postoperative parameters

As illustrated in Table 3, there were no significant differences in the occurrence or severity of PONV at 0–3 h postoperatively between the two groups; however, there was a significant reduction in PONV at 3–24 h postoperatively after OAP implementation (*P* = 0.005). In the CAP group, 38 patients (70.37%) experienced PONV compared to 21 (42.86%) in the OAP group (*P* = 0.006) (Table 3). Regarding the comparison of PONV severity, 20 (37.04%) patients in the CAP group and six (12.25%) in the OAP group complained of moderate-to-severe PONV (*P* = 0.006) (Table 3). Regarding postoperative antiemetic use, the two groups had similar antiemetic treatment and time-to-first-remedy (Table 4). In addition, there was no significant difference between the two groups regarding postoperative analgesia, tramadol dosage used in the PACU and ward, incidence of moderate-to-severe pain, and PLOS.

**Table 3:** Incidence and severity of PONV among the participants

	PONV severity	CAP group (*n* = 54)	OAP group (*n* = 49)	*P*
0–24 h PONV, *n* (%)				0.258
	0 (None)	7 (12.96)	11 (22.45)	0.299
	1 (Nausea)	9 (16.67)	13 (26.53)	0.240
	2 (Retching)	19 (35.19)	13 (26.53)	0.397
	3 (Vomiting)	19 (35.19)	12 (24.49)	0.285
	Moderate-severe PONV	38 (70.37)	25 (51.02)	0.068
0–3 h PONV, *n* (%)				0.675
	0 (None)	10 (18.52)	12 (24.49)	0.481
	1 (Nausea)	11 (20.37)	13 (26.53)	0.492
	2 (Retching)	18 (33.33)	13 (26.53)	0.522
	3 (Vomiting)	15 (27.78)	11 (22.45)	0.651
	Moderate-severe PONV	33 (61.11)	24 (48.98)	0.239
3–24 h PONV, *n* (%)				0.005
	0 (None)	16 (29.63)	28 (57.14)	0.006
	1 (Nausea)	18 (33.33)	15 (30.61)	0.834
	2 (Retching)	10 (18.52)	5 (10.21)	0.273
	3 (Vomiting)	10 (18.52)	1 (2.04)	0.009
	Moderate-severe PONV	20 (37.04)	6 (12.25)	0.006

Continuous data are expressed as mean ± standard deviation or median (interquartile range). Categorical data are expressed as the number of patients (percentage). Statistical significance was tested using the t-test, Mann–Whitney U test, Pearson’s chi-square test, and Fisher’s exact test

CAP, conventional anaesthetic protocol; OAP, optimised anaesthetic protocol; PONV, postoperative nausea and vomiting

**Table 4:** Postoperative variables

	CAP group (*n* = 54)	OAP group (*n* = 49)	*P*
Antiemetic treatment, *n* (%)	54 (100.00)	49 (100.00)	> 0.999
Antiemetic remedy treatment, *n* (%)	7 (12.96)	4 (8.16)	0.531
Time-to-first-remedy (h)	7.90 (7.86)	9.87 (10.65)	0.732
Postoperative analgesia, *n* (%)	8 (14.82)	6 (12.24)	0.779
Tramadol in the PACU (mg)	0 (0, 0)	0 (0, 0)	0.362
Tramadol in the ward (mg)	100 (0, 100)	100 (0, 100)	0.555
Moderate-severe pain (NRS ≥ 4), *n* (%)	2 (3.70)	0 (0.00)	0.496
PLOS (d)	5 (4, 5)	4 (4, 5)	0.142

Continuous data are expressed as mean ± standard deviation or median (interquartile range). Categorical data are expressed as the number of patients (percentage). Statistical significance was tested using the t-test, Mann–Whitney U test, Pearson’s chi-square test, and Fisher’s exact test

CAP, conventional anaesthetic protocol; NRS, numeric rating scale; OAP, optimised anaesthetic protocol; PACU, post-anaesthetic care unit; PLOS, postoperative length of stay

## Discussion

As demonstrated in the current RCT, compared with sevoflurane inhalation anaesthesia combined with conventional fluid management, propofol intravenous anaesthesia combined with GDFT decreased the incidence of PONV at 3–24 h postoperatively by 27.51%. Concerning the severity of PONV, OAP implementation primarily reduced the incidence of vomiting 3–24 h postoperatively but did not affect nausea and retching. There were no between-group differences in antiemetic treatment, awakening time, PACU time, incidence of moderate-to-severe pain, and PLOS.

Despite using various prophylactic strategies, including multimodal pharmacologic antiemetic agents and optimisation of anaesthesia schemes, the incidence of PONV within 24 h after LSG in our study was 78%. According to previous studies, anaesthesia-related factors affecting the incidence of PONV in bariatric surgery primarily include opioid use, inhaled anaesthetics, and perioperative fluid therapy [5, 12]. Reducing the use of opioids during the perioperative period can reduce the incidence of PONV in patients undergoing bariatric surgery but may be detrimental to postoperative pain management [13]. Total intravenous anaesthesia and optimised fluid management are the most easily implemented anaesthesia management strategies for patients undergoing LSG at our institution.

Evidence suggests that shortening the preoperative fasting duration and providing appropriate volumes of perioperative fluid replacement therapies can lower the incidence of PONV [14, 15]. Individualised GDFT optimises fluid therapy and improves outcomes in patients undergoing bariatric surgery [16]. Employing dynamic indices such as stroke volume variation, pulse pressure variation, and PVI to guide fluid therapy may play a crucial role in preventing excessive fluid administration in patients undergoing abdominal surgery, which could improve outcomes, such as enhancing recovery of gastrointestinal function, reducing PONV occurrence, and possibly shortening PLOS [17–20]. Therefore, the GDFT is a part of the OAP that decreased PONV in our study. However, there were no differences in total intra-operative infusion volumes between the two groups in our study. Our findings are inconsistent with those of previous studies [18–20]. There are several reasons for this discrepancy. First, fluid administration in the CAP group was determined partly at the discretion of the attending anaesthesiologist, which restricted fluid infusion. Therefore, the total volume infused in our control group was lower than that used in a previous study. Second, the preoperative fluid deficit was calculated based on the LWB, and the duration of surgery was short (< 3 h) in both groups, resulting in less total fluid infusion volume. In previous studies, the difference in fluid administration between the GDFT and standard care groups varied in numerous GDFT trials because of different fluid management protocols, haemodynamic parameters, and duration of the operation. A meta-analysis of 56 studies on GDFT reported that the differences were within 500 mL in 35 (62%), > 500 mL in 10 (18%), and <-500 mL in 11 (20%) trials [21]. The intra-operative colloidal fluid infusion was notably higher in the OAP group than in the CAP group. Colloid administration was more effective than crystalloid administration in preventing PONV in patients undergoing elective, noncardiac, or major surgery under general anaesthesia for > 3 h [22]. However, the role of colloid infusion in preventing PONV after LSG requires further investigation.

In this RCT, anaesthetic drugs majorly affected PONV because there was no statistical difference in the infusion volume between the two groups. Inhalation anaesthetics are reliable, independent predictors of PONV, whereas propofol has antiemetic properties and is associated with quicker recovery [23–25]. However, studies that compared the effects of TIVA and inhalation anaesthetics on PONV in bariatric surgery reported inconsistent results. A randomised controlled study found no significant difference in PONV between intravenous anaesthesia with propofol and desflurane in patients who underwent laparoscopic bariatric surgery [26]. However, another early RCT demonstrated that TIVA without the use of opioids was associated with a significant reduction in the incidence and severity of PONV compared to inhalation anaesthesia combined with opioids [27]. Furthermore, Elbakry et al. [6] found that TIVA using propofol and dexmedetomidine was associated with a lower incidence of PONV and better postoperative recovery than inhalation anaesthesia using desflurane. Another RCT discovered the combined effect of TIVA and multi-pharmacotherapy in reducing PONV scores without decreasing PONV-related delays to discharge [7]. In the present study, TIVA decreased PONV at 3–24 h but did not reduce the incidence and severity of PONV at 0–3 h postoperatively in patients undergoing LSG. What explains the difference in the effects of TIVA on the incidence of PONV at different time points after LSG? The multimode prophylactic anti-nausea drugs dexamethasone and azasetron were routinely used during the procedure to prevent patients from experiencing severe PONV and other adverse effects. Therefore, the anti-nausea effect of TIVA was not significant in the early postoperative period (0–3 h).

Our study had certain limitations. First, while the researchers who followed up with the patients and collected data were blinded to the groupings, it was not feasible to blind the attending anaesthesiologists who performed the interventions. Second, the influence of intraoperative anaesthesia strategy may be extensive and may not be limited to PONV. Studies should focus on the overall quality of recovery after surgery. The quality of this study could be improved if additional outcome measures (e.g., sleep quality, pain score, QOR15.) was included as a secondary outcome measure. Third, the optimization of intraoperative anaesthesia management strategy only included anaesthesia maintenance drugs and fluid management. If more parameters can be optimized, it would have greater clinical value for high-risk patients having nausea and vomiting.Finally, our study was monocentric and utilized a specific anaesthesia protocol, which could limit its applicability to other clinical settings or countries.

## Conclusions

Optimal anaesthesia strategy was associated with a significantly lower incidence and severity of PONV in patients undergoing LSG. Using TIVA instead of a volatile anaesthetic is an effective multimodal antiemetic strategy for bariatric surgery.

## Data Availability

The data associated with the paper are not publicly available but are available from the corresponding author on reasonable request.

## Abbreviations

ASA: American Society of Anaesthesiologists
BIS: Bispectral index
BMI: Body mass index
CAP: Conventional anaesthetic protocol
ERAS: Enhanced recovery after surgery
GDFT: Goal-directed fluid therapy
LBW: Lean body weight
MAP: Mean arterial blood pressure
NRS: Numerical rating scale
OAP: Optimised anaesthetic protocol
PACU: Post-anaesthetic care unit
PLOS: Postoperative length of stay
PONV: Postoperative nausea and vomiting
PVI: Pleth variability index
RCT: Randomised controlled trial
SD: Standard deviation
TIVA: Total intravenous anaesthesia
